# Total Hip Arthroplasty with Subtrochanteric Femoral Shortening Osteotomy for Crowe Type IV Post-Dysplastic Hip Osteoarthritis: Clinical and Radiological Outcomes

**DOI:** 10.3390/jcm15072685

**Published:** 2026-04-02

**Authors:** Marek Rovnak, Marian Melisik, Maros Hrubina, Jozef Cabala, Juraj Cabala, Martin Feranec, Zoltan Cibula

**Affiliations:** 1 Jessenius Faculty of Medicine in Martin, Comenius University Bratislava, 036 59 Martin, Slovakia; marek.rovnak@uniba.sk (M.R.); marian.melisik@uniba.sk (M.M.); martin.feranec@uniba.sk (M.F.); 2 Department of Orthopaedic Surgery, University Hospital Martin, Kollarova 2, 036 59 Martin, Slovakia

**Keywords:** developmental dysplasia of the hip (DDH), total hip arthroplasty (THA), femoral shortening osteotomy

## Abstract

**Background:** Surgical management of adult patients with post-dysplastic coxarthrosis using total hip arthroplasty is technically demanding and carries an increased risk of complications. In cases of high iliac dislocation classified as Crowe type IV, restoring the acetabular component to the anatomical hip centre often requires femoral shortening osteotomy to enable safe reduction in the prosthetic joint. Nevertheless, long-term evidence on functional outcomes and prosthesis survival with this approach is limited. **Methods:** A retrospective cohort study included 19 patients with 22 cases of Crowe type IV post-dysplastic hip osteoarthritis treated with uncemented total hip arthroplasty (Pinnacle/S-ROM, DePuy, Warsaw, IN, USA) combined with transverse subtrochanteric femoral shortening osteotomy. Patients underwent serial clinical follow-up, including assessment of range of motion, measurement of limb-length discrepancy, and functional evaluation using the Harris Hip Score and the WOMAC questionnaire. Radiological assessment included evaluation of osteotomy union, implant positioning, and osteolysis on standardized radiographs. Vertical distances of the centre of rotation (CR), the tip of the greater trochanter (GT), and the tip of the lesser trochanter (LT) from both reference lines were measured bilaterally, and inter-side differences were calculated. The reference lines consisted of the line connecting the inferior margins of the ischial bones and the teardrop (TD) line. **Results:** All osteotomies united at a mean of 5.57 months, with a mean follow-up of 129 months. Mean limb-length discrepancy decreased from 5.27 cm to 1.5 cm, and mean hip flexion improved from 82.9° to 106°. Functional outcomes improved significantly, with mean WOMAC increasing from 55.4 to 80.1 (*p* < 0.001) and mean Harris Hip Score from 49.8 to 84.66 at up to 3 years of follow-up (*p* < 0.001). Osteotomy length correlated strongly with lesser trochanter–teardrop distance (*p* = 0.00000048). Complications included distal femoral fissure (27.3%) and revision (18%), with no infection or permanent neurological deficit. **Conclusions:** Total hip arthroplasty combined with subtrochanteric femoral shortening osteotomy for Crowe type IV post-dysplastic hip osteoarthritis appears to be a feasible and effective procedure in an experienced centre, providing reliable osteotomy healing and significant early functional improvement that is sustained over time. Limb-length discrepancy was reduced and satisfactory biomechanical restoration was achieved, with an acceptable complication profile and implant survival of 81.3% at long-term follow-up. The LT–TD parameter was identified as a potential predictor of osteotomy length, enabling the proposal of a preoperative planning equation. However, given the limited sample size and lack of validation, these findings should be interpreted cautiously. Further studies are needed to confirm their broader applicability.

## 1. Introduction

Developmental dysplasia of the hip (DDH) is a spectrum of anatomical abnormalities involving the femoral head and the acetabulum. This condition encompasses a range of pathological states of the hip joint, from acetabular dysplasia through subluxation to complete dislocation. Normal acetabular development depends on the growth of the triradiate cartilage epiphysis, which arises from three ossification centres within the acetabular portions of the pubis, ilium, and ischium. Furthermore, the position and presence of the spherical femoral head within the acetabular socket are crucial for stimulating physiological acetabular development.

According to several authors, DDH is the most common congenital defect in newborns [1]. Osteoarthritis secondary to DDH is the most frequent cause of secondary hip osteoarthritis and therefore warrants increased clinical attention [2]. Surgical management of adult patients with post-dysplastic coxarthrosis using total hip arthroplasty (THA) is often technically demanding and carries an increased risk of complications, requiring experience and a thorough understanding of the altered anatomical relationships.

In cases of high iliac dislocation classified as Crowe type IV, restoring the acetabular component to the anatomical hip centre often requires a femoral shortening osteotomy to enable safe reduction in the prosthetic joint. This THA implantation is technically challenging and associated with a higher complication rate than standard THA performed for primary osteoarthritis [3].

Several levels of femoral osteotomy may be performed, including proximal, distal, and subtrochanteric shortening osteotomies. Subtrochanteric shortening osteotomy can be performed using various configurations, such as transverse, oblique, step-cut (Z-shaped), or V-shaped techniques. A meta-analysis, which included 37 studies comprising 795 hips, reported no significant differences among subtrochanteric osteotomy techniques in terms of non-union rate, revision rate, nerve injury, dislocation, or achieved Harris Hip Score. Consequently, transverse subtrochanteric osteotomy is commonly recommended and performed as a simple and effective technique with favourable clinical outcomes [4].

## 2. Materials and Methods

This retrospective cohort study included patients with post-dysplastic hip osteoarthritis classified as Crowe type IV who underwent total hip arthroplasty (THA) with transverse subtrochanteric femoral shortening osteotomy between January 2009 and December 2018 at a single tertiary referral centre.

All procedures were performed with patients supine, using an anterolateral surgical approach. In all cases, an uncemented hemispherical press-fit acetabular component (Pinnacle, DePuy, Warsaw, IN, USA) was implanted at the anatomical hip centre. For intraoperative orientation in determining the centre of rotation, we used bony prominences and flat areas in the area of original acetabulum in accordance with preoperative planning. For some more difficult cases, we had prepared a sterile 3D printed model of the patient’s pelvis based on a preoperative CT scan.

Acetabular cup sizes ranged from 42 to 52 mm. We tried to implant the largest shell size as possible for larger insert diameter and better stability. We have used ceramic-on-ceramic articulation pairing for long-term implant survival and low wear. In case of insufficient press fit of the shell or insufficient bone coverage, we used 2 screws for additional fixation, depending on the surgeon’s perioperative decision. We did not use bone grafts, cotyloplasty or augmentations. A modular uncemented femoral stem (S-ROM, DePuy, Warsaw, IN, USA) was used in all patients. A transverse subtrochanteric femoral shortening osteotomy was performed in every case to enable safe joint reduction, restore limb length, and minimize tension on neurovascular structures. First, the femoral canal and proximal sleeve were prepared. The femoral canal was pre-drilled to the diameter of the definitive implant. The sleeve orientation was directed toward the area with the greatest femoral bone stock. The osteotomy was performed solely for shortening and not for derotation.

Before performing the osteotomy, the rotational alignment was marked and transferred to the distal fragment. The osteotomy level was approximately 1 cm below the level of the trial sleeve and the lesser trochanter, which was determined intraoperatively using fluoroscopy. The resected femoral segment was approximately 5 mm shorter than that planned preoperatively. A trial reduction was subsequently performed.

Before insertion of the definitive implant, a prophylactic cerclage band was placed around the proximal portion of the distal osteotomized fragment to reduce the risk of periprosthetic fracture. The implant design provides excellent primary and rotational stability of the osteotomy due to the proximal cold-weld connection between the sleeve and the stem and the double longitudinal slot of the distal stem. In addition, the intraoperative variability of the implant allows reconstruction of the altered anatomical proportions of the dysplastic hip. Furthermore, the implant design enables postoperative compression at the osteotomy site.

The study included patients aged 14–68 years. Although the inclusion of a pediatric patient is unusual for this indication, the adolescent patient was treated using the same protocol as the adult patients. Inclusion criteria were applied consistently across all age groups.

Postoperatively, all patients followed a standardized rehabilitation protocol, patients relieved the operated hip joint using French crutches for 3 months. Full weight bearing was allowed after complete consolidation of the osteotomy

Clinical evaluation was performed preoperatively and at 3, 6, 12 and 36 months postoperatively, with subsequent follow-up visits at two-year intervals. Hip joint range of motion (ROM) was measured with a goniometer. Limb length discrepancy (LLD) was assessed clinically by measuring the distance between the anterior superior iliac spine and the medial malleolus. Functional outcomes were evaluated using the Harris Hip Score (HHS) and the Western Ontario and McMaster Universities Osteoarthritis Index (WOMAC), with these assessments routinely conducted within a follow-up period of up to three years. HHS was assessed by the treating physician and categorized as poor (<70 points), fair (70–80 points), good (80–90 points), or excellent (90–100 points). WOMAC scores were converted to a percentage scale (0–100%), with higher values indicating better functional status, and categorized as poor (<43%), fair (43–55%), good (56–75%), or excellent (>75%).

**Figure 1:**
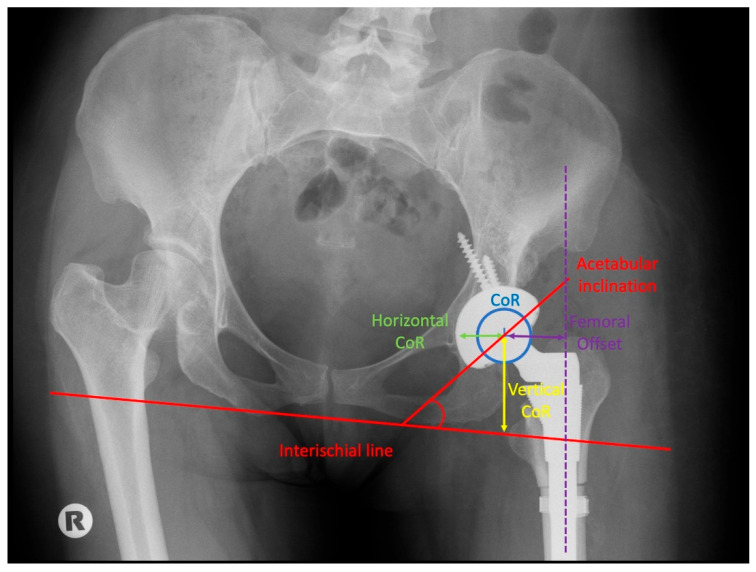
Postoperative radiographic assessment of implant position: horizontal and vertical centre of rotation, acetabular inclination, and femoral offset.

Radiological assessment included evaluation of osteotomy healing, osteolysis and implant positioning, including vertical and horizontal centre of rotation, femoral offset and acetabular inclination. (Figure 1).

Osteotomy union was defined by callus formation, restoration of cortical bone continuity, and the gradual disappearance of radiolucent lines at the osteotomy site. Osteolysis of the femoral component was assessed using Gruen zones, while acetabular osteolysis was evaluated using DeLee and Charnley zones.

**Figure 2:**
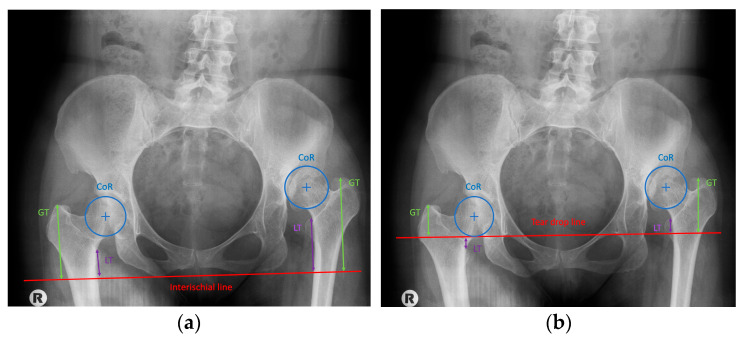
Measurement of radiological point from the reference line of the ischial bones (**a**); measurement of radiological point from the reference line of tear drop (**b**).

**Figure 3:**
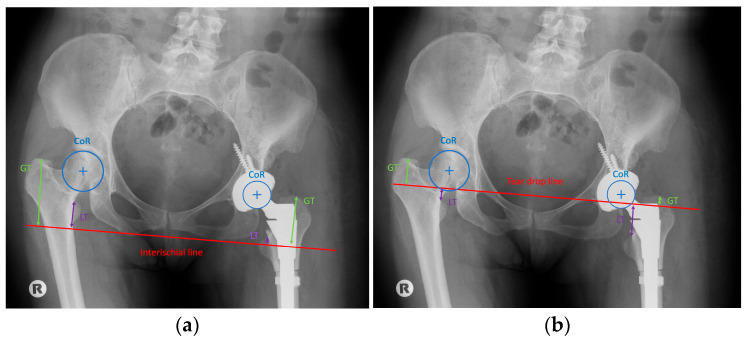
Postoperative measurement of radiological point from the reference line of the ischial bones (**a**); postoperative measurement of radiological point from the reference line of tear drop (**b**).

For preoperative planning, radiographic measurements were obtained from anteroposterior pelvic radiographs using two reference lines: the line joining the inferior margins of the ischial bones and the teardrop (TD) line. Vertical distances from both reference lines to the centre of rotation (CR), the tip of the greater trochanter (GT), and the tip of the lesser trochanter (LT) were measured bilaterally, and inter-side differences were calculated. To ensure the clinical validity and reproducibility of the radiographic parameters, all measurements were independently performed by two orthopedic surgeons (M.F., J.C.) both of whom were blinded to the intraoperative resection data. Standardized anteroposterior pelvic radiographs were obtained with the patient in the supine position and both hips internally rotated by 15° to neutralize femoral antetorsion; neutral pelvic orientation was strictly verified by the symmetry of the obturator foramina and the precise vertical alignment of the coccyx with the symphysis pubis. All digital imaging analyses and calibrations were executed within the hospital Picture Archiving and Communication System (PACS). Preoperative magnification was corrected using a 25 mm radiopaque calibration sphere, while postoperative measurements were calibrated based on the known diameter of the prosthetic femoral head. The study cohort comprised 19 patients, including 16 with unilateral and three with bilateral Crowe IV dysplasia. In the 16 unilateral cases, the contralateral healthy hip served as the primary anatomical reference for inter-side difference calculations. For the three bilateral cases, where a physiological contralateral template was absent, a theoretically restored anatomical centre of rotation (CR)—established using the pelvic teardrop and ischial markers—was utilized as the baseline. To evaluate the robustness of the methodology, intra-observer and inter-observer reliability were statistically assessed using the Intraclass Correlation Coefficient (ICC), which demonstrated excellent agreement (ICC = 0.90). Correlation and linear regression models were subsequently employed to identify the most accurate predictor and to formulate a robust equation for estimating the required osteotomy length. (Figure 2 and Figure 3).

A paired Student’s *t*-test was used to compare preoperative and postoperative continuous variables, such as limb length discrepancy (LLD) and hip flexion improvement, for simple pre–vs–post comparisons. Changes in HHS, WOMAC, and LLD over multiple time points were analyzed using a linear mixed model (LMM), with time as a fixed effect and patient ID as a random effect to account for repeated measurements. The Wilcoxon signed-rank test was applied to analyze changes in range of motion (ROM).

Pearson or Spearman correlation analysis was used to evaluate relationships between continuous variables (e.g., age, BMI, and hip flexion) depending on data distribution. Correlations between these parameters and intraoperatively resected osteotomy length were analyzed using correlation and regression models. Linear regression analysis was performed to identify the most accurate predictor and to develop a formula for estimating the required osteotomy length. Model fit was evaluated using R^2^ and adjusted R^2^. The slope coefficient (β) and its 95% confidence interval were reported. Normality of residuals was assessed using the Shapiro–Wilk test.

Implant survival was analyzed using Kaplan–Meier survival analysis, with revision surgery for any reason defined as the primary endpoint. Patients who had not undergone revision by the time of final follow-up were considered censored observations.

Paired comparisons of radiographic parameters between early postoperative and final follow-up measurements were performed using either the paired Student’s *t*-test or the Wilcoxon signed-rank test, depending on data distribution.

Statistical significance was set at *p* < 0.05.

## 3. Results

This retrospective cohort study included 19 patients with Crowe type IV post-dysplastic hip osteoarthritis who underwent subtrochanteric femoral shortening osteotomy combined with total hip arthroplasty (THA). The cohort comprised 16 women and three men, with a total of 22 THA procedures performed concurrently with shortening osteotomy. Sixteen patients underwent unilateral surgery, while three underwent staged bilateral procedures. The mean age at surgery was 49 years (range, 14–68 years), and the mean body mass index (BMI) was 26.95 kg/m^2^ (range, 17.51–34.67 kg/m^2^). Fourteen left hips and eight right hips were operated on. The mean follow-up duration was 129 months (range, 20–188 months). (Table 1).

The mean length of hospital stay was 9 days (range 7–12 days). All 22 osteotomies achieved union without evidence of pseudoarthrosis. The mean time to radiographic union was 5.57 months (range 3–12 months).

Among patients with unilateral dysplasia, the mean preoperative limb length discrepancy (LLD) was 5.27 cm (range 2–14 cm), decreasing postoperatively to a mean of 1.5 cm (range, 0–5 cm), a statistically significant improvement (*p* = 0.000214). The mean lengthening of the operated limb was 3.2 cm. The mean osteotomy length was 4.56 cm (range, 1.5–7 cm).

Mean hip flexion improved from 82.86° preoperatively to 105.95° at 12 months postoperatively, demonstrating a statistically significant improvement (*p* = 0.000146). The magnitude of improvement showed moderate negative correlations with age (r = −0.49) and BMI (r = −0.45), suggesting reduced functional gain in older patients and those with higher BMI. At final follow-up, the mean range of motion included flexion of 105.9°, abduction of 35.5°, adduction of 23.6°, internal rotation of 20.7°, and external rotation of 28.2°. (Table 2).

WOMAC and Harris Hip Scores (HHS) demonstrated significant improvements postoperatively (Table 3). Linear mixed model analysis, accounting for repeated measures within patients, showed a rapid functional improvement within the first 3 months after surgery.

WOMAC score increased from 55.4 ± 21.8 at baseline to 74.7 ± 11.3 at 3 months, 76.0 ± 13.11 at 6 months, 80.1 ± 14.7 at 12 months, and 80.1 ± 17.7 at 36 months.

HHS increased from 49.8 ± 14.5 at baseline to 69.4 ± 9.3 at 3 months, 78.0 ± 11.7 at 6 months, 84.2 ± 13.3 at 12 months, and 84.66 ± 12.02 at 36 months.

All postoperative improvements compared to baseline were highly significant (*p* < 0.001). Pairwise comparisons between postoperative time points indicated no statistically significant differences beyond 3 months, suggesting functional gains stabilized after the first postoperative year. The largest effect sizes occurred between baseline and 3 months (WOMAC +18.56, 95% CI 11.31–25.82; HHS +19.6, 95% CI 13.0–26.2). The linear mixed model accounted for patient-level random effects (ICC = 0.42) and explained 27.5% of marginal variance and 57.9% of conditional variance for WOMAC scores.

These results indicate that THA with transverse subtrochanteric femoral shortening osteotomy provides rapid and sustained improvements in hip function, with gains largely achieved within the first 3 months and maintained at long-term follow-up.

Radiographic measurements are summarized in Table 4. The mean early postoperative vertical centre of rotation was 20.38 ± 9.9 mm compared to 20.12 ± 9.74 mm at final follow-up (*p* = 0.709), and the mean horizontal centre of rotation was 28.17 ± 2.68 mm compared to 27.20 ± 2.69 mm (*p* = 0.150). The mean femoral offset was 37.76 ± 5.11 mm versus 37.90 ± 4.99 mm (*p* = 0.821), and the mean acetabular inclination was 50.82 ± 6.76° versus 50.40 ± 7.13° (*p* = 0.559). None of the evaluated parameters demonstrated statistically significant differences.

Radiographic analysis in Table 5. showed the strongest correlations between osteotomy length and measurements referenced to the teardrop line. The highest correlation was observed for the distance from the lesser trochanter to the teardrop line (Pr(>|t|) = 0.00000048), followed by the distance from the centre of rotation to the teardrop line (Pr(>|t|) = 0.000001). Measurements referenced to the ischial line showed weaker correlations.

Linear regression analysis demonstrated that the LT_TD difference significantly predicted osteotomy length. The resulting regression equation was osteotomy length = 1.6104 + 0.4757 × LT_TD difference.

**Figure 4:**
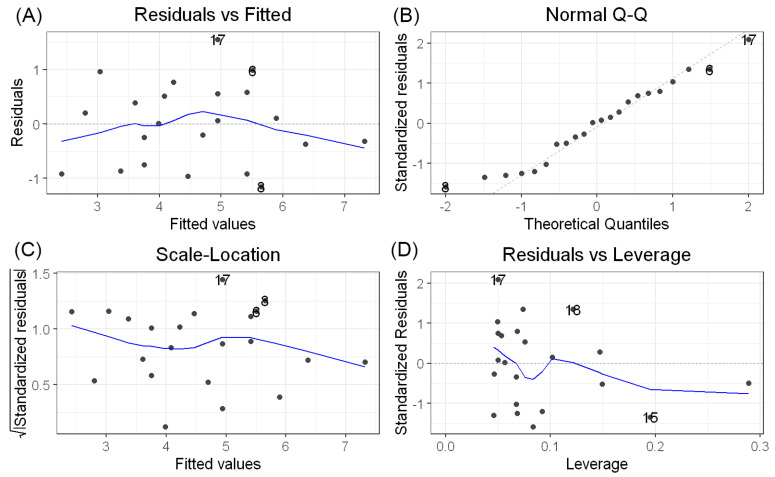
Regression diagnostic plots for the linear model predicting osteotomy length based on the LT_TD difference. (**A**) residuals vs. fitted values, (**B**) normal Q–Q plot, (**C**) scale–location plot, and (**D**) residuals vs. leverage.

The model explained 72.6% of the variance in osteotomy length (R^2^ = 0.726; adjusted R^2^ = 0.713) and was statistically significant (F(1,20) = 53.07, *p* < 0.001). The slope coefficient for LT_TD difference was significant (β = 0.4757, SE = 0.0653, *p* < 0.001), with a 95% confidence interval ranging from 0.35 to 0.60. Residual diagnostics confirmed normal distribution of residuals according to the Shapiro–Wilk test (W = 0.964, *p* = 0.565). Figure 4.

The most accurate linear model for estimating osteotomy length was:

osteotomy length = 1.6104 + 0.4757 × (LT_TD difference)

Intraoperative distal femoral fissures occurred in six cases (27.3%) and were managed with cerclage fixation without further sequelae. Revision surgery was required in four cases (18%). There were two revisions for aseptic loosening femoral component (9.1%). In the first case, due to loosening of the stem of the femoral component with presumed insufficient stem diameter and rotational instability, the well-integrated sleeve was removed and a new proximal sleeve and a long, one size bigger—15 mm/235 mm diameter—revision stem was implanted. In the second case, revision was performed a year and a half after the initial implantation due to loosening of the entire femoral component, including the sleeve with radiolucent zones in all zones. A revision modular stem with distal fixation was used due to bone defects of the proximal femur. Two patients were revised for recurrent dislocation (9.1%). In the first patient, dislocations occurred 4 months after the initial implantation. During the revision, the stem was changed from 36 mm to 42 mm of the same diameter, leaving the sleeve in place, and a 12 mm head was implanted to obtain sufficient stability. The dislocation in the second patient occurred 6 months after THR and was caused by loosening and vertical migration of the acetabular component. The patient underwent revision of the acetabular component with reimplantation and fixation using two screws, combined with Harris acetabuloplasty and a structural bone graft. The femoral stem was reoriented with the sleeve left in place, and the femoral head was exchanged for a longer one. Intraoperative and postoperative complications are summarized in Table 6.

**Figure 5:**
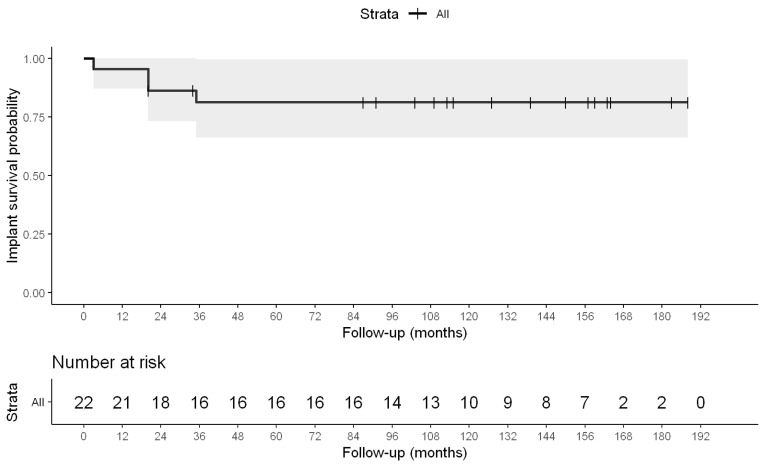
Kaplan–Meier survival curve (implant survival).

Implant survival was further analyzed using Kaplan–Meier analysis. The estimated implant survival probability was 81.3% at an average of 129 months. Four patients need revision procedures during the follow-up period. The Kaplan–Meier survival curve with the corresponding risk table is presented in Figure 5 and Table 7.

No infections, deep vein thrombosis, or permanent neurological deficits were observed. Heterotopic ossification (Brooker II) was identified in one case (4.5%) without clinical symptoms. Radiographic osteolysis of ≤1 mm was observed in two cases (9%) without clinical correlation. Osteolysis of the femoral component was identified in 1 of 22 patients, involving Gruen zones 1 and 4. Acetabular osteolysis was observed in 1 patient, affecting DeLee and Charnley zones 1, 2, and 3. No other cases of osteolysis were detected during the follow-up period.

All osteotomies achieved successful union, and overall clinical and radiological outcomes showed functional improvement with an acceptable complication rate in this challenging patient population.

## 4. Discussion

This retrospective analysis evaluated long-term outcomes in patients who underwent transverse subtrochanteric femoral shortening osteotomy during implantation of an uncemented modular femoral stem. The primary objectives were to assess clinical outcomes using WOMAC and Harris Hip Score (HHS), evaluate postoperative radiographic findings, and compare intraoperative and postoperative complication rates with previously published data. Additionally, by performing radiographic measurements and comparing anatomical landmarks and reference lines on standard radiographs, we aimed to propose an optimal formula for estimating osteotomy fragment length. Such preoperative planning is considered essential for achieving favourable postoperative outcomes and minimizing perioperative complications.

The mean osteotomy length in our cohort was 4.56 cm, comparable with previously reported values of 2.5 to 4.5 cm [5,6]. Although increasing osteotomy length has been associated with a higher risk of non-union [7], no such cases were observed in our series. The mean postoperative limb lengthening was 3.2 cm, which we consider safe with respect to the risk of neurological injury. This finding aligns with published data suggesting that limb lengthening of up to approximately 4.5 cm can be achieved without significant neurological complications [8].

The use of cemented femoral stems in transverse subtrochanteric osteotomy has been associated with a higher prevalence of non-union [9] and prolonged healing time compared with uncemented fixation [10]. Reported healing times for uncemented stems range from 4 to 5.5 months [5,11]. In our cohort, an uncemented modular S-ROM stem was used in all cases, with a mean osteotomy healing time of 5.57 months, consistent with the literature.

Functional outcomes showed substantial improvement after surgery. Three years postoperatively, the mean WOMAC score was 80.1%, and the mean HHS was 84.66%. Similar favourable HHS outcomes (81–94%) have been reported by other authors, with follow-up periods ranging from 36 to 120 months [8,12,13]. These findings support the effectiveness of THA combined with subtrochanteric shortening osteotomy in restoring function in patients with severe developmental dysplasia.

Radiographic assessment of limb length discrepancy traditionally relies on two pelvic reference lines: the teardrop line and the ischial line. In our analysis, measurements referenced to the teardrop line showed significantly stronger correlations with osteotomy length than those referenced to the ischial line. This observation aligns with previous reports favouring the teardrop reference because of the variability of the ischial line associated with pelvic rotation [14,15].

Commonly used proximal femoral landmarks include the greater trochanter, the centre of rotation of the femoral head, and the lesser trochanter. Previous studies comparing these anatomical reference points in individuals without significant anatomical abnormalities found no significant differences [16,17], consistent with our findings. However, in our cohort, the highest correlation was observed for measurements obtained from the lesser trochanter, closely followed by those from the centre of rotation. Therefore, these two landmarks appear most suitable for accurate preoperative planning. Notably, more distal femoral reference points showed stronger correlations, which may reflect the substantial anatomical variability of the proximal femur in DDH.

Regarding imaging modalities, computed tomography has not been shown to offer significantly greater measurement accuracy than standard radiographs for this purpose [18]. Given radiation exposure and resource utilization, conventional radiographic measurements appear sufficient for most patients.

Reported complications of THA with subtrochanteric osteotomy include intraoperative femoral fractures, prosthetic dislocation, osteotomy non-union, neurovascular injury, and implant failure [19]. In our cohort, intraoperative fissures of the distal femoral fragment occurred in six hips (27.3%), comparable with reported rates of 10% to 28% [20,21]. Recurrent dislocation requiring revision occurred in two patients (9%), consistent with previously published rates [8]. Radiographic osteolysis was observed in one acetabular and one femoral component (9%), whereas the literature reports rates of 7% to 43% for uncemented implants in long-term follow-up [22,23].

To optimize fragment contact and promote union, osteotomy height relative to the lesser trochanter may be adjusted according to osteotomy length. Previous authors have reported improved fragment contact when osteotomy was performed 2 cm below the lesser trochanter for fragment lengths up to 2.5 cm, and approximately 1.5 cm below for fragments measuring 3–5.5 cm [24]. In our cohort, osteotomies were routinely performed 2–3 cm distal to the reference line, regardless of fragment length, and no non-union was observed.

During long-term follow-up, revision surgery was required in 4 of 22 hips, yielding an implant survival rate of 82%. Comparable long-term survival rates of 79% to 96% have been reported in similar patient populations [24,25]. Earlier studies using older polyethylene acetabular liners reported higher revision rates due to wear and osteolysis, with survival at 67% after 19 years [26]. Implant longevity may also be influenced by patient age at surgery, with lower survival observed in younger patients, likely reflecting higher activity levels [27].

The major limitation of this study is the relatively small sample size (19 patients, 22 hips). Although comparable to previously published case series on this procedure, the limited number of cases may reduce the statistical power of the analysis and increase the risk of selection bias, thereby limiting the generalizability of the findings. This limitation reflects the rarity and complexity of the studied surgical indication. Nevertheless, a strength of the present study is the long-term follow-up, which provides valuable insight into the outcomes of this demanding reconstructive procedure.

Furthermore, while our cohort included a high proportion of unilateral cases (16/19), the application of the formula in the 3 bilateral Crowe IV cases required the use of a virtual anatomical baseline. Although our results remained consistent, we recognize that a larger cohort of bilateral cases is necessary to further validate the universal applicability of this predictive model.

## 5. Conclusions

Total hip arthroplasty combined with subtrochanteric femoral shortening osteotomy for Crowe type IV post-dysplastic hip osteoarthritis is a technically feasible and reproducible procedure in an experienced centre. The technique achieved a 100% osteotomy union rate, with a mean time to union of 5.6 months, confirming its reliability.

The procedure resulted in rapid and clinically meaningful functional improvement, with the greatest gains observed within the first 3 months postoperatively and sustained over time, as demonstrated by WOMAC and HHS outcomes. Limb-length discrepancy was significantly reduced, and satisfactory restoration of hip biomechanics was achieved without significant radiographic changes at long-term follow-up.

The complication profile was acceptable for this complex patient population, with manageable intraoperative fissures and a revision rate of 18%, primarily due to femoral loosening and instability. Kaplan–Meier analysis demonstrated an implant survival of 81.3% at a mean follow-up of 129 months.

Radiographic analysis identified the LT–TD parameter as a relevant predictor of osteotomy length, enabling the formulation of a practical equation for preoperative planning. This approach may represent a useful and clinically applicable concept in the management of complex dysplastic hips. However, given the limited sample size and the absence of internal and external validation, these findings should be interpreted with caution. The potential impact of measurement error, as well as the restricted generalizability beyond the specific implant, surgical technique, and institutional setting, should also be considered. Further studies are warranted to validate and refine these proposed relationships.

Overall, this study supports the effectiveness of this technique in achieving stable long-term outcomes while providing a structured and clinically applicable approach to preoperative planning in severe hip dysplasia.

## Figures and Tables

**Table 1:** Age, follow-up, and BMI in patients undergoing femoral shortening osteotomy with THA for severe developmental hip dislocation.

Variables	Overall (Hips)	Ø	S	Min	Max
Age	22	49	15	14	68
Duration of follow-up in months	22	129	45	20	188
BMI (kg/m^2^)	22	26.95	5.06	17.51	34.67

BMI—Body mass index.

**Table 2:** Mean range of motion at final follow-up.

Variables	Mean ROM FFU
Flexion	105.9°
Abduction	35.5°
Adduction	23.6°
Intra-rotation	20.7°
Extra-rotation	28.2°

**Table 3:** Linear mixed model of WOMAC and Harris Hip Score (HHS) over 36 months.

Time Point	WOMAC Score (Mean ± SD)	Estimate (95% CI)	*p*-Value	HHS (Mean ± SD)	Estimate (95% CI)	*p*-Value
0 months	55.4 ± 21.8	48.6–62.1	<0.001	49.8 ± 14.5	44.2–55.4	<0.001
3 months	74.7 ± 11.3	+18.6 (11.3–25.8)	<0.001	69.4 ± 9.3	+19.6 (13.0–26.2)	<0.001
6 months	76.0 ± 13.1	+20.55 (13.2–27.9)	<0.001	78.0 ± 11.7	+28.2 (21.0–35.4)	<0.001
12 months	80.1 ± 14.7	+25.5 (18.1–33.0)	<0.001	84.2 ± 13.3	+34.4 (27.0–41.8)	<0.001
36 months	80.1 ± 17.7	+26.0 (18.5–33.55)	<0.001	84.66 ± 12.02	+34.86 (27.4–42.3)	<0.001

WOMAC score—Western Ontario and McMaster Universities Osteoarthritis Index, HHS—Harris hip score.

**Table 4:** Postoperative radiographic assessment of the horizontal and vertical centre of rotation, acetabular inclination, and femoral offset.

Variables	Mean (Early Postoperative)	*p*-Value	Mean (Last Follow-Up)
Veritical CoR	20.38 ± 9.90	*p* = 0.709	20.12 ± 9.74
Horintal CoR	28.17 ± 2.68	*p* = 0.150	27.20 ± 2.69
Femoral offset	37.76 ± 5.11	*p* = 0.821	37.90 ± 4.99
Acetab. inclination	50.82 ± 6.76	*p* = 0.559	50.40 ± 7.13

**Table 5:** Results of separate univariate linear regression models, each including osteotomy length as the dependent variable and one radiographic parameter as the independent variable.

Variables	Estimate	Std. Error	t-Value	*p*-Value
CR	0.572169	0.096365	5.937528	0.000008
GT	0.356937	0.076257	4.680692	0.000144
LT	0.447335	0.073599	6.077964	0.000006
GT-TD	0.406294	0.071881	5.652287	0.000016
CR-TD	0.644856	0.094871	6.797190	0.000001
LT-TD	0.475702	0.065301	7.284750	0.00000048

CR—Centrum of Rotation, GT—Tip of the Greater Trochanter, LT—Tip of the Lesser Trochanter, TD—Tear Drop.

**Table 6:** Intraoperative and postoperative complications.

Variables	Overall	Percentage
Intraoperative complication		
Fissure of the distal femoral fragment	6/22	27.3%
Postoperative complication		
Revision	4/22	18%
Femoral loosening	2/22	9.1%
Hip prosthesis dislocation	2/22	9.1%
Heterotopic ossification	1/22	4.5%
Osteolysis	2/22	9.1%
Deep venous thrombosis	0/22	0%
Neurological complication	0/22	0%
Infection	0/22	0%

**Table 7:** Kaplan–Meier survival analysis demonstrated implant survival of 81.3% at average of 129 months. Four revision procedures occurred during the follow-up period.

Time	n_risk	n_event	Survival	Lower_CI	Upper_CI
3	22	1	0.9545	0.8714	1.000000
20	21	2	0.8636	0.7315	1.000000
35	17	1	0.8128	0.6627	0.99697

## Data Availability

The original contributions presented in this study are included in the article. Further inquiries can be directed to the corresponding author.

## Abbreviations

The following abbreviations are used in this manuscript:

DDH	Developmental Dysplasia of the Hip
THA	Total Hip Arthroplasty
LLD	Limb Length Discrepancy
WOMAC	Western Ontario and McMaster Universities Osteoarthritis Index
HHS	Harris Hip Score
TD	Teardrop
CR	Centre of Rotation
GT	Greater Trochanter
LT	Lesser Trochanter
ANOVA	Analysis of Variance
BMI	Body Mass Index
